# Technology Games: Using Wittgenstein for Understanding and Evaluating Technology

**DOI:** 10.1007/s11948-017-9953-8

**Published:** 2017-08-15

**Authors:** Mark Coeckelbergh

**Affiliations:** Department of Philosophy, University of Vienna, Universitätsstraße 7 (NIG), 1010 Vienna, Austria

**Keywords:** Wittgenstein, Technology, Language, Language games, Technology games, Form of life, Philosophy of technology, Social robots

## Abstract

In the philosophy of technology after the empirical turn, little attention has been paid to language and its relation to technology. In this programmatic and explorative paper, it is proposed to use the later Wittgenstein, not only to pay more attention to language use in philosophy of technology, but also to rethink technology itself—at least technology in its aspect of tool, technology-in-use. This is done by outlining a working account of Wittgenstein’s view of language (as articulated mainly in the *Investigations*) and by then applying that account to technology—turning around Wittgenstein’s metaphor of the toolbox. Using Wittgenstein’s concepts of language games and form of life and coining the term ‘technology games’, the paper proposes and argues for a use-oriented, holistic, transcendental, social, and historical approach to technology which is empirically but also normatively sensitive, and which takes into account implicit knowledge and know-how. It gives examples of interaction with social robots to support the relevance of this project for understanding and evaluating today’s technologies, makes comparisons with authors in philosophy of technology such as Winner and Ihde, and sketches the contours of a phenomenology and hermeneutics of technology use that may help us to understand but also to gain a more critical relation to specific uses of concrete technologies in everyday contexts. Ultimately, given the holism argued for, it also promises a more critical relation to the games and forms of life technologies are embedded in—to the ways we do things.

## Introduction

The empirical turn in the philosophy of technology (Achterhuis 2001) is and was a turn to artefacts, things. Consider for instance the seminal work of Don Ihde (1990; 1998), a material hermeneutics and phenomenology of technology, or Peter-Paul Verbeek’s work on ‘what things do’ (Verbeek 2005). This turn to things was an understandable and helpful move in the field, which helped to bridge humanities and engineering approaches to the philosophy of technology, to use a distinction made by Carl Mitcham (1994). However, the turn was also a turn away from language. What seems missing in a contemporary philosophy of technology is a systematic account of the relationships between language and technology (Coeckelbergh 2015, 2017b). Moreover, in the philosophy of language, plenty of work has been done on what we do with words, often inspired by Austin (1962), Searle (e.g. 1975, 1995), or indeed Wittgenstein (e.g. 1953). However, far less attention has been paid to what we do with things, and how this relates to our use of language. In this programmatic and explorative paper, it is proposed that we remedy this gap between the philosophy of technology and the philosophy of language by drawing on the later work of Ludwig Wittgenstein, with a focus on the *Philosophical Investigations* (1953) and, to a lesser extent, *On Certainty* (1969). In particular, Wittgenstein’s view of language is used and applied. My initial idea was that a better understanding of our use of language, along Wittgensteinian lines, can help us to develop an argument about the relation between technology and language.

However, my proposal in this paper is not merely “adding” an understanding of *language* to the philosophy of technology, for instance in the form of analysing language we use to talk *about* technology. The aim of the proposed approach and programme is more ambitious and further-ranging: it is proposed to use Wittgenstein to re-think the philosophy of *technology*. As a first step in this direction, which already shows at least *one* way in which we can render Wittgenstein’s thinking fruitful for thinking about technology, it is proposed to use and modify the concepts of language games, form of life, and grammar as conceptual tools to better understand *technology*. In particular, the concept of “technology games” is proposed and it is argued that we can interpret form of life and grammar in a way that offers a use-oriented, holistic, transcendental, normative, social, and historical understanding of technology, understood here as tool. There is also an emphasis on implicit knowledge as opposed to interpretations that only consider rule-following.

The structure of my paper is as follows. First a working account and interpretation of Wittgenstein’s view of language is offered: his focus on use and his use of technology as a metaphor to understanding language is outlined, a holistic and transcendental interpretation is offered, and the dimensions of normativity and implicit knowledge are highlighted. Then this working account of Wittgenstein’s view of language is applied to thinking about the use of technology – with technology understood as a tool, as technology-in-use. Turning around Wittgenstein’s toolbox metaphor, it is argued that the use of *technology* can be helpfully and meaningfully understood in a Wittgensteinian manner: in a holistic, transcendental, and normative way, and as involving implicit knowledge next to more explicit, formal and theoretical kinds of knowledge. In line with Wittgenstein’s own focus on use in the everyday lifeworld, it is shown what this means for concrete technologies by giving examples from the domain of (social) robotics.

## Wittgenstein on Language: A Use-Oriented, Holistic, and Transcendental Working Interpretation

There is a long and rich reception history of Wittgenstein’s work in philosophy, and it is impossible to do justice to it here. For the purposes of this paper, a summary and interpretation of what Wittgenstein says about language is offered by drawing on the *Philosophical Investigations* and *On Certainty*, and on the way some references are made to similar interpretations in order to clarify my working account. At least the following claims about language can be detected:

First, according to Wittgenstein in the *Philosophical Investigations* (1953), meaning depends on use. The meaning of a word is not attached to word-objects, but depends on how we use the word. Use gives a sign its ‘life’ (Wittgenstein 1953, §432, p. 135e). For Wittgenstein, language is an instrument: ‘Language is an instrument. Its concepts are instruments.’ (§569, p. 159e). Words, then, are like tools. Interestingly, at this crucial point of his argument, Wittgenstein uses a technological metaphor:

‘‘Think of the tools in a toolbox: there is a hammer, pliers, a saw, a screwdriver, a rule, a glue-pot, glue, nails and screws. – The functions of words are as diverse as the functions of these objects. (And in both cases there are similarities.)’ (Wittgenstein 1953, §11, p. 9e)

Words, then, can have various meanings, depending on their use. Wittgenstein relates the use of language to various activities: language is woven into our activities (§7, p. 8e). He gives the examples of giving orders, describing something, acting in a play, etc. (§23, p. 15e). The meaning of words depends on these use-contexts. This can be compared to Heidegger’s point in *Being and Time* that ‘a sign is not really “comprehended” when we stare at it (…)’; signs refer to ‘what is actually “going on.”’ (Heidegger 1927, p. 74).

This takes us to the next feature of Wittgenstein’s view of language: holism. For Wittgenstein, use of language depends on larger wholes, for which he uses the concepts ‘language games’ and ‘form of life’. He writes:

‘‘I shall also call the whole, consisting of language and the activities into which it is woven, a “language-game”.’ (Wittgenstein 1953, §7, 9e)

Hence for Wittgenstein language is not a separate realm of signs, a distinct domain of symbols. Language must be understood in its use and is hence interwoven with our activities and games, which each have their own rules. How we use words depends on how we do things in a particular activity and game. This is in turn related to how we live, to a larger whole Wittgenstein calls a ‘form of life’:

‘to imagine a language means to imagine a form of life.’ (Wittgenstein 1953, §19, 11e)

Metaphorically speaking, activities, language games and forms of life can thus be seen as a kind of concentric “circles” (2D metaphor) or “spheres” (3D metaphor) that surround and shape a particular use of language. Although these metaphors do little justice to the temporal, process character of use, activities, and games, let me compare their relations by means of the following diagram:

According to this interpretation, the use of language must be seen as embedded in activities and structured by games and a form of life, which contains many games. Other interpretations would equate language games and form of life; I do not object to such an interpretation, but then one would need another concept to express that there is a whole that contains language games. There are of course many discussions about the precise relation between language games and forms of life among interpreters of Wittgenstein (see for instance Whiting 2017). For the purposes of this paper, it is important to stress that language use is holistically related to games and forms of life. Holism here means that it is impossible to understand the use of language without understanding the activities, games, and forms of life in which it is embedded; and vice versa, a form of life means nothing apart from the uses, activities, and games. The form of life only lives in use. Meaning thus stretches across all concentric circles. Moreover, the form of life could be called a “culture”, which then in turn contains particular games and activities. Indeed, this interpretation is in line with the “cultural” interpretation of Wittgenstein, for instance that of Winch (1958), who focused on rule-following.

However, with this cultural interpretation come a number of dangers. First, in line with Wittgenstein it must be stressed that culture should not be reified and externalized; as said culture only lives in use and activities. Therefore, using the concept ‘form of life’, rather than “culture”, seems more appropriate since it enables us to stress *lived* language: language only makes sense, literally, in use and activities. (Indeed it might be said that like Merleau-Ponty emphasized the lived body, Wittgenstein emphasized lived language.) Second, the social and cultural life should not be reduced to rule-following, if only because not all knowledge about how to use language and how to do things can be reduced to explicit knowledge; there is also implicit knowledge – what Polanyi called ‘tacit’ knowledge. (This point will be revisited.) Third, the claim that culture shapes particular uses of language and hence particular meanings should not be interpreted as a quasi-causal claim that has the form of “A shapes B” in the sense of “A causes B to change form”. The use of language is not “caused” by culture and language games and forms of life should not be interpreted as external things, let alone agents that can cause something.

In order to avoid the latter interpretation, I propose to give a transcendental interpretation to language games and forms of life. In his *Critique of Pure Reason* (1781), Kant famously used the phrase “conditions of possibility” (German: *Bedingungen der Möglichkeit*) of experience and knowledge. His idea was that our experience is “made possible” and structured by categories. They provide limits to what we can know. Going beyond Kant, however, the transcendental method can be used in order to interpret Wittgenstein and more specifically to define the relation between on the one hand language use and its meaning, and on the other hand language games and form of life. Wittgenstein’s claim about language then becomes not only the general one that language use is “related to” language games and forms of life, but the relation is specified as follows: language games and forms of life are transcendental conditions of language use and meaning in particular situations. They “make possible”,^1^ structure, and limit a particular use of language and its related meaning. Without these games and forms of life, a particular use of language would be meaningless. Another way of saying this is to use the term “grammar”: a particular use of language is made possible by words but also by a grammar that is given, that is already there before a particular use of language.

This interpretation of Wittgenstein is in line with for instance the interpretation offered by Gier (1980), who argued that forms of life ‘perform a transcendental function’ in the sense that they are the patterns in our lives ‘that make a meaningful world possible’ (Gier 1980, p. 257). It can also be directly supported by Wittgenstein’s own claim that his inquiry is transcendental and grammatical:

‘yet our investigation is directed not towards phenomena, but rather, as one might say, towards the ‘possibilities’ of phenomena. […] Our inquiry is therefore a grammatical one.’ (PI § 90)

Furthermore, my use of the word grammar can also be supported by Wittgenstein’s distinction between ‘surface grammar’ and ‘depth grammar’. The latter is not something that we can easily explain (make explicit), but it must be presupposed to make sense of our use of language.

‘In the use of words, one might distinguish ‘surface grammar’ from ‘depth grammar’. What immediately impresses itself upon us about the use of a word is the way it is used in the sentence structure, the part of its use – one might say – that can be taken in by the ear. – And now compare the depth grammar, say of the verb “to mean”, with what its surface grammar would lead us to presume. No wonder one finds it difficult to know one’s way about.’ (Wittgenstein 1953, § 664, pp. 176e-177e)

I propose to interpret this passage as implying that our use of language is not only shaped by a particular surface grammar as syntax, which includes rules concerning how to compose a sentence, but also by a depth grammar in the form of games and a form of life, which constitutes a transcendental condition that must be presupposed in order for the sentence to have meaning.

Note that this use of the term ‘transcendental’ does not imply any suggestion of the ‘transcendence’ (as opposed to immanence) of grammar. The grammar of language use does not exist “above” or “apart” from the concrete uses of language and the activities they are connected to. It is also not “in the head” or in a “noumenal” realm. If anywhere, it “is” and lives in concrete use, in human activities and practices. (If Wittgenstein’s perspective must be characterized by using the immanent/transcendent dichotomy at all, then it is rather immanent.)

To this picture of Wittgenstein’s view of language the following two dimensions must be added. The first is a normative one. The rules of games and the meanings and values that are part of a form of life are not only descriptive; they are also normative. They tell us what to do: they tell us how to use language and how to do particular activities. If I utter a sentence, I *have* to follow grammar (surface grammar); I cannot compose my sentence as I wish, my composition is not entirely optional. There are grammatical rules, rules of syntax. However, there is also a ‘depth’ grammar which is not so easy to formalize. In my use of language there will be also normative meanings “slipping in” that are part of the game and part of our culture. For instance, when people hear the word “surgeon”, they often assume that the surgeon is male. This is made possible by meanings that are around in our culture and form of life: historically and in Western culture this profession was often done by men, and there is a gender bias around in our society. There is thus a depth grammar that shapes a particular use of language, and which is normative.

Furthermore, not all knowledge involved here is explicit, as in the case of rules – which are not always formulated but *can* always be made explicit, in principle. As already indicated, there is also implicit knowledge and knowledge that is not theoretical but has the mode of know-how: knowing language is not only and – if Wittgenstein’s use-oriented view is followed – not mainly about having theoretical knowledge; rather, it is a about *knowing how* to use language. Moreover, not every grammar can be formalized in rules. Perhaps surface grammar, syntax can be formalized. But the depth grammar is less easy if not impossible to fully make explicit. There is a lot that we presuppose, a lot that makes possible a particular use of language. There is a lot of implicit knowledge about how to do things with words and more generally how to do things.

In *On Certainty* (1969), Wittgenstein argues that in daily life such implicit knowledge is enough. He writes: ‘the squirrel does not infer by induction that it is going to need stores next winter as well. And no more do we need a law of induction to justify our actions and our predictions.’ (§287, p. 37e) And he gives a very instructive example of taking hold of a towel: we do this ‘without having doubts’ (§510, p. 67e). Similarly, knowing how to use language is about knowing how to ‘do certain things’ (§534, p. 71e). It is about learning activities and games.

This implies that Wittgenstein’s view of language is also social. The knowledge of activities and games is shared with others. Language use is part of our doing things with others. This view is in tune with Dewey’s understanding of language in *Experience and Nature*: it is an ‘instrument of social cooperation and mutual participation’, a tool that enables social participation (Dewey 1929, p. vi). It is also about doing things and getting others to do things. And in line with what later Austin will call the ‘perlocutionary’ force of language, Dewey writes that language is ‘a mode of action used for the sake of influencing the conduct of others in connection with the speaker’ (p. 206). Again the view of language as a tool and language-in-use leads us to its normative uses.

Finally, it must be added that use of language and the games and form of life that shape it are historical: use of language changes and there is a given, but this given always changes – if only slightly. If use of language is always related to activities, then use may change as activities change. Language games and forms of life also change, although as individuals (i.e. individual language users and individual gamers) our influence on them is limited. This also means that normativity changes and evolves; however, this kind of change too, is slow and not entirely up to individuals or individual use. This historical and “flux” aspect of language and forms of life can be nicely captured by using the metaphor of a river-bed Wittgenstein uses in *On Certainty*: just as ‘the river-bed of thoughts may shift ‘, but only partly and often imperceptibly (Wittgenstein 1969, §§97-99, p. 15e), so language and its structures and normativities may shift over time. Wittgenstein stresses that we inherit a picture of the world (§94, p. 15e) and we inherit a language. As our language use sometimes cuts new banks, change is possible. But this change may be imperceptible and slow; the banks that guide the river are relatively stable, some of the rocks are hard. When we learn language, there is already a river-bed, there is already a language and a form of life. As Stern puts it, ‘at any given time, one must take some things for granted, and that taken for granted background limits what one can say and do.’ (Stern 1995, p. 190) Thus here is a balance between flux (change) and stability.

To conclude this section, Wittgenstein offers a use-oriented, holistic, social, historical, and transcendental view of language, which has normative and implicit aspects, and which enables us to critically study and comment on language in its concrete uses, embedded in language games and forms of life. And perhaps we can try to make explicit these games and forms of life, or at least hint at them, in order to be able use language in a more critical way. To use a famous metaphor that is often applied to Wittgenstein’s method: philosophy can be done as “therapy”.

Of course much more can be said about this and other aspects of Wittgenstein’s philosophy of language. However, it is not this paper’s purpose and project to offer a comprehensive interpretation of Wittgenstein’s view of language or to engage in a Wittgensteinian philosophy of language as such. I *use* Wittgenstein in this paper. (Note also that, Wittgenstein and I have *used* language to make the point that language is about use – thus demonstrating the very point in and by writing, understood as language use. Indeed, any writing about language is done *in and by language*; it is thus itself a kind of *use*.) Based on this brief but *use-full* outline and working view of language, the second part of this paper can now be started, which is not about language but about *technology*.

## Using Wittgenstein for Thinking About Technology

One potential application of Wittgenstein’s view of language to the philosophy of technology is employing Wittgenstein’s view and method to focus on the use of language, including its normative dimension: analysis of the *use of language* in discourses about technology, and critical discussion of the “technology games” in the sense of “language games concerning technology” – perhaps applying Wittgensteinian “therapy” to language use in the philosophy of technology. Indeed, language use *about* technology can be critically studied: language use by philosophers of technology and by various (other) users of technology. Such a study could be “therapeutic” in the sense that, by focusing on how people use language concerning technology, some common misunderstandings and confusions about technology may be revealed (e.g. the conception of technologies as mere instruments). Or one may study the “politics” of language games concerning technology. I believe this is a very important project and also important with regard to the politics of technology, as Langdon Winner has argued in a recent keynote.^2^ Moreover, here on the focus is limited to the later work of Wittgenstein, but of course it is also possible to read other parts of Wittgenstein’s work and to try to render it fruitful for the philosophy of technology. Consider for instance Alfred Nordmann’s work on the *Tractatus* (e.g. Nordmann 2002). Another project could be to further reveal and discuss various places in which Wittgenstein talks about technology and/or to focus on Wittgenstein’s background as an engineer. For example, Susan Sterrett (2005) has argued that Wittgenstein’s view of language in the *Tractatus* is influenced by his experiences with technologies in his youth and his work as an engineer.

However, here my inquiry is limited to the following operation and project: to apply Wittgenstein’s view of the use of *language* (as found mainly in the *Investigations*) to the use of *technology*. For this purpose, let me return to Wittgenstein’s toolbox:

‘‘Think of the tools in a toolbox: there is a hammer, pliers, a saw, a screwdriver, a rule, a glue-pot, glue, nails and screws. – The functions of words are as diverse as the functions of these objects. (And in both cases there are similarities.)’ (Wittgenstein 1953, §11, 9e)

In this passage Wittgenstein compares the use of words to the use of tools in order to say something about language. But I propose to turn the metaphor around: instead of comparing language to technology, we can explore what it would mean to compare *technology* to language – with language understood in the Wittgensteinian sense summarized in the first part of this paper. What happens if what Wittgenstein says about language use is applied to the use of technology, understood as tool?

First, as this question already indicates, a use-oriented view of technology is obtained. While arguably not *all* meanings related to a particular technology can be reduced to those connected to its use (and hence the present project may not be *sufficient* for a comprehensive theory of technology) and technology can have many more meanings and forms than that of a tool (e.g. network, infrastructure, process, etc.), this orientation captures a very important and indeed *necessary* aspect of technology and one of its meanings (tool). Without use, technology as tool would be meaningless. It is use that gives technology life. A use orientation, which as said is also present in Ihde and more recently and from another angle has been proposed by Franssen and Koller (2016), has the advantage that it, in Wittgensteinian form, directs us to concrete, everyday uses of technology, to what we may call *lived* technology, technology-in-use. This could also support Ihde’s claim that a technological object becomes what it is ‘through its uses’ (Ihde 1990, p. 70); hence the meaning of the technology is multistable, it depends on the context (p. 144).

Second, however, I propose a specific understanding of this use, in particular the holistic and transcendental version outlined above. Technologies, considered in their use, are part of activities and games. What may be called “technology games” shape and give meaning to particular uses. Particular uses of technology are made possible by games and forms of life. They are only meaningful and use-full on the basis of these transcendental conditions, which structure and limit them.

Let me further unpack this. How we use technologies is shaped by the games and forms of life that are already in place “before” we use them. There is already a “grammar” of technology. Of course there is also a “grammar” in the sense of “syntax”: specific rules how to put together different parts for instance, or specific operating instructions. But there is also a grammar in a wider, more social and cultural sense: there are already particular activities and ways we do things, there are already games, and the technologies are part of those games and their use is shaped by the games. For our use to be possible and for technological artefacts to be meaningful, these games and – ultimately – a form of life must be presupposed as transcendental conditions. And again “transcendental” does not mean “transcendent”: in this interpretation, the term “transcendental” refers to what must be presupposed, but this does not take us to another world; the transcendental conditions only live, only play out, in concrete uses of technology and concrete technological practices.

This understanding of technology as being shaped by its use in various activities and games (as transcendental conditions that structure and limit) is not only a specific use and interpretation of Wittgenstein; it can also be supported by how Heidegger’s formulates the default understanding of tools in *Being and Time*: usually, tools are experienced as ready-to-hand (*Zuhandenheit*). He gives the example of a hammer: we encounter the hammer as ‘a useful thing’, and as such it withdraws; its handiness is discovered in the activity of hammering, not theoretically (Heidegger 1927, p. 65). In other words, usually technologies are experienced *in use*; they are not present (outside use). And that use is related to, and refers to, a world in which users live, that is, ‘our world’, which is a ‘public world’ alongside the world of nature (Heidegger 1927, p. 66). Thus, while what Heidegger calls ‘useful things’ are of course also material and refer to other material things, their meaning can only be fully understood in reference to a larger whole that is human, lived, public, social, and cultural, and which we always presuppose when we talk about things and when we use things. More, using Wittgenstein it can be said that this larger whole, that form(s) of life *structures* use, provides a *grammar* for use.

To understand what this approach means for understanding technologies, consider examples of interaction with so-called “social robots”: robots designed for, and used in, social (or one may say: quasi-social) interaction. On the one hand, what Wittgenstein says about language use can be applied to our talking about and talking to such robots: next to surface grammar which structures our composition of our sentences, what we say is made possible by language games and forms of life – larger depth grammars. For instance, if we talk about robots as slaves, then this involves an entire culture and form of life, with a particular history in which slavery has played a role. On the other hand, it can also be said that there are “technology games”, that the use of *technology* – and not merely the use of language – is also shaped, structured, and made possible by games and forms of life. Their may be operating instructions for using the robot. But there is also a much “deeper” or “wider” kind of grammar, which has to do with the way we do things, with our games and our form of life. The use of, and interaction with, the social robot is connected to particular activities and games, such as meeting someone, drinking coffee, talking about one’s health, asking a pet to do something, cuddling a pet, and so on, which each are (part of) social games with particular rules and know-how that come with them. Our interactions follow the rules and grammar of social relations and social games. Without such social games, the very use of a “social” robot would not be possible; the games constitute a transcendental condition. At the same time, the transcendental conditions and the grammar do not exist outside of the concrete uses and practices (i.e. as if there was a thing “culture” separate from use, activities, etc.); it is concrete use, activity, and practice that give life to the grammar. It is in concrete use that the transcendental conditions play out.

Note that this holistic and grammatical analysis does not only work in the case of social robots and other technologies that can actively interact with us and speak with us, such as for instance a navigation device in a car, where the meaning of the device is entirely connected to its use in the context of specific activities and games of driving and wayfinding (grammars concerning how to operate the device but also grammars of how we do things in a certain context, e.g. how we drive). It is also applicable to more thing-like cases such as hammers and coffee cups. To take up again the (Wittgensteinian and Heideggerian) example of a hammer: it is impossible to understand the meaning of a hammer without understanding the uses and activities of hammering, which are embedded in specific games such as repairing or maintaining something (e.g. a shed or a roof) – games which are in turn connected to other specific uses and activities (e.g. activities related to living in a house or gardening activities) and to an entire socio-technological environment in which use of roofs, gardens, and houses are part of *the way we do things*. Technology games are both technological and social, and as transcendental (but not transcendent, since related to concrete practices and material artefacts) conditions they make possible and structure specific uses of technology.

Moreover, such technology games are not to be understood as entirely distinct from language games: the games involve both the use of language and the use of tools. Technology games are also language games. For instance, as Wittgenstein knew building involves specific uses of language, for instance use of the word “Slab!”(e.g. Wittgenstein 1953, §6, p7e). It is a technological, social, and linguistic game. And vice versa, language games are also technology games. For instance, having coffee with someone involves speech but also the handling of things such as a cup, a spoon, perhaps a coffee machine, etc. (And indeed speech may itself be seen, in a Wittgensteinian manner, as a kind of technology use: the use of words.) Technology games, then, are always at the same time also social games and language games. Our use of technology goes together with (and co-constitutes) our social and cultural practices, and these always involve language to some extent for coordination and communication. The social life almost always involves the use of words and the use of (other) tools. Language and technology interconnect and are entangled in their use and in games. Moreover, these activities and games are in turn part of a larger form of life/culture/world, which shapes the often implicit meanings that govern the games. Technologies are part of a form of life, part of what we do and how we do things; they are part of what we are.

This form of life is normative in the sense that it shapes and structures our expectations and interactions, understood as use, activities, and games. It does not “cause” or “determine”, but is holistically interconnected with particular uses of words and things. The form of life lives in use, and use has a form that goes beyond the particular use and situation. Furthermore, the forms and games constitute a kind of implicit knowledge that is usually not doubted. Wittgenstein writes in *On Certainty*: ‘If I make an experiment I do not doubt the existence of the apparatus before my eyes’ (Wittgenstein 1969, §337, p. 43e). Similarly, one could say that usually we do not doubt the technologies we use and when we have implicit know-how, we already know how to use them. For instance, once we are used to a particular game such as meeting someone, we know what to do when we “meet” a social robot; the same social-language-technology game applies. We already have the know-how, we already know-how; we have implicit knowledge based on social experience and games in other contexts and in relation to other people and other technologies. We already have rules and norms. We already know how to do things.

This interpretation of technology in terms of a ‘form of life’ is compatible with Winner’s application of the term ‘form of life’ to technologies: technologies are part of a form of life in the sense that they are ‘woven into the texture of everyday existence’ (Winner 1986, p. 12); life would be unthinkable without them. Even new technologies, such as (social) robots (my example) or computers (Winner’s example), then, are variations of older patterns (pp. 12-13) and shape our expectations, for example about computers (p. 14). The same could be said about robots: when we encounter a social robot, we have expectations. These expectations are linked to games and patterns that were already there before the particular encounter, and even before the robot was developed. For example, in the context of the language/social game “meeting someone” or “exchanging names”, we have the expectation that when we tell our name, the other person also tells her or his name. When the robot which resembles a social conversation partner does not meet that expectation (for instance because it lacks the necessary social-linguistic interactive intelligence), we may be disappointed. This also shows again how *social* our games are – apart from being linguistic and technological. Language, technology, and the social cannot be disentangled once we view them from this Wittgensteinian use-oriented and holistic angle.

However, in contrast to Winner’s earlier work on this and certainly in contrast to Ihde, who did not use Wittgenstein and who rejects any transcendental interpretation since he assumes it is necessarily abstract and non-empirical (see also Smith’s criticism of Ihde, Smith 2015), I offer a *transcendental* interpretation of form of life as condition of possibility that shapes and makes possible our use of technology. The term “culture” was already used, but this may be misleading: a form of life and grammar is not a “culture” in the sense of something external, but lives in the use of technology, makes possible the use of technology, and shapes the use of technology. This interpretation is better able to avoid the reification and externalisation of “culture”, keeping it alive so to speak; it also avoids any (quasi-)causal interpretation of the relation between culture and technology, as for instance in determinism and anti-determinism concerning technology or culture. To say that technology is part of a culture or form of life should not be understood as implying that there is an external thing “culture” that causes or determines “technology”, or vice versa. Understood holistically, the use of technology is part of and constitutes a form of life; the latter means nothing without the former and the former means nothing without the latter. Moreover, in line with Winner’s point about expectations but going further, the *normative* dimension of forms of life and technology games must be stressed. The grammars of technology are holistic, transcendental, historical, normative, and implicit. These aspects will now be further shown and elaborated.

Normativity can come in the form of explicit rules. But usually the normativity of technology games and forms of life is not visible and not made explicit. It exerts its influence on particular uses of technology (and the uses of language connected with those uses) in the form of an invisible grammar that gives meaning and norms. Consider again the example of social robots. When someone kicks a robot (consider for instance the case of robot dog Spot who was kicked by its developers^3^) and some people respond that this is wrong, then both the kicking and the response may be puzzling at first sight, given that the robot is supposed to be a “thing” or a “machine”. But with the approach proposed here, we can try to make explicit the normative structures that shapes particular uses. Here for instance it may be argued that this interaction and this response is structured and made possible by grammars of human-animal (and maybe even human-human) relations, which are already there before this happens. There is a form of life, which has a history, including violence towards (non-)humans and empathic responses to such violence. The same can be said in cases when robots are treated as “slaves”: both the use of the word “slave” and the use of technology as “slave” are embedded and made possible by a form of life in which slavery made (makes?) sense and was (is?) practiced. (Note that comparisons between technology and slavery are common, today and also in the history of philosophy of technology, from Aristotle who in the *Nicomachean Ethics* compares tools to lifeless slaves, to for instance Simondon’s comparison between recognizing the mode of existence of technical objects and the abolition of slavery – see Simondon 1958, p. 1) And when robots are meant to work in the household and when they are designed or imagined as a woman, then this real or imagined use could only happen, and is made possible, by gender meanings and gender relations and patterns that are already there before the particular design, manifestation, and use happened. The technology grammar is related to wider social and cultural grammars, which clearly have a normative dimension. Like our use of language, our use of technology enters and follows a river-bed that was already there before us and before our particular use. When we play a particular technology game and engage in a particular activity with technology and with others, we inherit these wider meanings and these historical patterns and “have” to follow them – this is the normativity. A particular technology grammar today is always related to older technology grammars. For instance, how to use the technology may be similar to how to use an older technology (grammar as syntax) and older meanings may come with the new technology-as-used (depth grammar), for example when past discussions about the harmfulness of watching TV (e.g. Postman 1985) transfer to discussions about use of internet-related technologies such as computers and smartphones. Another example: as I have recently argued (Coeckelbergh 2017a), our use of contemporary technology is still influenced by romanticism, which (as we can now reformulate) acts as a “grammar” with a history that still shapes and makes possible how we use and think about technology today. This also has normative implications, for instance if we use a particular understanding/construction of romanticism as a norm to reject or embrace new technologies, but also less directly when our thinking about machines is structured by modern and romantic oppositions. As I show in *New Romantic Cyborgs* (2017a), when we see machines as opposite of the human or when we try to merge with machines, these visions and desires have complex relations to modern-romantic thinking. We cannot simply leave the river-bed of modernity and its romantic banks.

Thus, to understand our use of, and interaction with, technology and for that use and interaction to make sense, we must presuppose that there are these patterns – games, forms of life, grammars – that make possible our use, interaction, and performance with the technology, that structure it – also normatively. One task for a phenomenology and hermeneutics of technology, then, is to reveal these grammars. This can be a step towards a more critical task. There is the given, but we can do more than revealing it and accepting it: we can also criticize the grammars. The transcendental approach then, in combination with recognizing the normativity of the grammars, is not only more “empirical” than has been supposed by Ihde, Verbeek and others in philosophy of technology who took an empirical turn, but also gives us a *critical* tool. By revealing and critically discussing the technology games and other grammars of technology, we can point not only to “the given” but also do the philosophy of technology in a critical way. Robots can be talked about in ways that relate them to gender issues, question human-animal relations, and so on. This “cutting into new banks” – by means of new language uses and new technology uses – is possible once we abandon atomistic and individualistic understandings of technology and move – with Wittgenstein and others – towards a more holistic and transcendental view of technology. In so far as it is a tool, technologies are embedded in larger structures and wholes, and next to studying particular uses, interactions and mediations, we should also reveal and discuss these larger structures and wholes that have a hermeneutic and normative force as they shape particular uses of technologies, understood as tools.

While in the current ethics of technology there is attention to, and recognition of, the normativity of technology and the social context of technology in the sense that technology shapes and “scripts” morality (Verbeek 2006 on “materializing morality”) and in the sense that values feed into the design process (see for instance value sensitive design as a response to this, e.g. Friedman et al. 2006: values should explicitly be taken into account when designing new technologies), the proposed approach offers an understanding of the normativity and sociality of technology that complements these existing approaches by asking the question regarding the structure and “grammar” of the normative whole – thus going beyond revealing particular mediations by artefacts and interactions in use and design processes and beyond identifying the normative context of use in terms of isolated and reified “values”. Instead, a focus on games and form of life suggests that particular mediations by particular artefacts are part of games and forms of life that exceed what happens at the level of the phenomenology and hermeneutics of individual use and interaction, or rather, that connects this phenomenology and hermeneutics to larger wholes and structures at the level of practices (games) and cultures (forms of life). For instance, in order to understand the normativity of a robot, it is not sufficient to talk about what a particular material artefact “does” in terms of shaping how we see the world and what we do as individual users. It is important to understand that robot in the context of games and a form of life that preceded the (use of the) particular artefact and the particular robot, games and a form of life that make possible and shape the robot’s meaning and normative consequences. These grammars are not about how to design, build, or operate the robot, but about how we live (together). Furthermore (and therefore), the transcendental and holistic approach enables us not only to be critical of a particular (use of) technology, but also of the larger structures in which it is embedded: the technology games and language games that are being played, and ultimately the entire form of life that makes possible the particular use, interaction, and mediations.

Now this could be interpreted as offering a “cultural” interpretation. This is not incorrect, but “culture” should not be seen as something external to use and games, including the use of technology and technology games. The proposed approach conceptualizes the normativity whole in terms of social and normative structures that shape and rule concrete use, but these structures and wholes exist in turn only as and in lived use. For instance, if there are such things as “values” at all and if they are part of our “culture”, then these values only have normative and semantic significance as part of forms of *life* and as part of everyday activities and games; that is, they only have meaning and only exist in use; values only exist when they are lived. For instance, the value of gender equality does not exist and is meaningless apart from concrete uses of language and technology, and the specific games that are played in practice. If and in so far as the “applied ethics” of technology suggests otherwise, it is vulnerable to the Wittgensteinian objection that concrete, everyday use comes first, and that we must reject any moral metaphysics (or “transcendent” norms) if such a metaphysics entails that moral principles, values, norms, and so on are meaningful outside our use of language, our use of technology, our language games, our technology games, and our form(s) of life. Moreover, the proposed transcendental interpretation of form(s) of life enables us to question “materializing morality” and value sensitive design approaches in so far as they assume that users and designers of technology have a large degree of control over the normative implications of their use and design. Instead, the proposed view acknowledges the normative force of the given, of the games and forms of life in which our use of technology (and hence also design of technology, understood as a kind of use) takes its course. While not denying the possibility and reality of change, this position avoids the suggestion of a voluntaristic model of technological and normative change, according to which such change is simply a matter of our human individual or collective will and actions to bring about change, e.g. through a different design. Things may be designed and used differently, of course, but some rocks we encounter are harder than others and change will take time. Particular artefacts may be re-designed, but we cannot simply re-design technology use, technology games, and the forms of life in which these are embedded.

Similarly, on the one hand it must be acknowledged that the relation between technologies and games/forms of life goes both ways. Technologies are not only embedded in forms of life; they also shape forms of life. They can be *game changers*. Perhaps many current information and communication technologies play such a role: they changed and are changing how we do things. For instance, e-mail has changed the way we work. Once again it becomes clear that technologies can have normative influence. On the other hand, however, in order to properly analyse these more grammatical changes and often subtle normative influences, one must first understand the precise ways in which the technologies are already embedded in, and entangled with, pre-existing games and forms of life. This approach promises to enable a more holistic, structural, and comprehensive analysis than for instance current mediation theory can offer (for more engagement with postphenomenology see Coeckelbergh 2017b).

To conclude, we can discern three important tasks of a (post?)^4^ Wittgensteinian holistic, transcendental, and critical phenomenology and hermeneutics of technology. First, it is to reveal the technology games and other “grammars” of technology (understood here as technology-in-use, technology as tool): not only the surface grammar of how technologies are used, composed, and operated, but also the games and the form(s) of life that structure and make possible our current uses of technology. Second, it should reveal the *normativity* in and of those grammars, and critically reflect on the games and forms of life in which our technologies – understood in terms of the use of tools – are embedded. Third, it is to show how technologies – understood not as isolated artefacts but as artefacts-in-use embedded in activities, games and a form of life – can indeed function as *game changers* and may slowly but surely change the river-beds of our form of life. These games, normativities, and game changes can then be critically evaluated. But first the technologies (and languages) need to be studied and their grammars need to be revealed. The proposed approach is not at all opposed to an empirical turn. All three tasks require not only conceptual work but also (or rather at the same time) empirically sensitive investigations into our use of language and technology, words and things.

## Conclusion

If there is a gap in the philosophy of technology with regard to thinking about language, then the use of Wittgenstein proposed in this paper may help to close it. It can do so by drawing our attention to language use concerning technology. However, this programmatic and explorative paper has also proposed a more ambitious research program influenced by Wittgenstein: I have argued that using Wittgenstein can also help us to re-think technology itself in a more holistic and transcendental way. Applying Wittgenstein’s view of language to technology, this paper has offered the concepts of technology games, forms of life, and grammar as tools we can use for moving in that direction. I proposed a holistic, transcendental, social, and critical phenomenology and hermeneutics of technology use that discusses technologies – understood, after the empirical turn, as technological artefacts – in the context of the technology games, form of life, and other grammars that make possible and structure that use. These games and forms of life *may* in turn be changed by the use of technologies, albeit slowly and often invisibly. This holistic and grammatical approach, which of course needs further development (for instance by means of more engagement with existing approaches in the philosophy of technology and by connecting to debates in recent Wittgenstein scholarship), thus assists us to gain a critical relation to specific uses of technologies. Some examples of how this works out for understanding and evaluating concrete technologies have been offered, and it has been argued that the approach can helpfully complement existing approaches in the field. However, ultimately and given the holism of the approach, the result and aim should go beyond criticizing specific technologies and their use; it should also involve criticizing the games and forms of life themselves. According to the holistic approach proposed in this paper, thinking about technology is also thinking about the ways we do things, and ultimately about our world and an entire form of life.

## Footnotes

1 I am aware that this is not good English but I will nevertheless use the phrase “make possible” throughout this manuscript, keeping “make” and “possible” together in order to refer to this specific (post)Kantian meaning.

2 Workshop “Wittgenstein and Philosophy of Technology”, University of Vienna 13 March 2017. https://philtech.univie.ac.at/research/events/past-events/workshop-wittgenstein-and-philosophy-of-technology-13032017/.

3
http://edition.cnn.com/2015/02/13/tech/spot-robot-dog-google/.

4 It is post-Wittgensteinian in the sense that it is no longer directed at language use but at language use *and* technology use (and their relation). It is fully Wittgensteinian, however, in its approach.
